# Mitochondrial Role in Intrinsic Apoptosis Induced by a New Synthesized Chalcone in Hepatocellular Carcinoma Cells

**DOI:** 10.3390/biomedicines10123120

**Published:** 2022-12-02

**Authors:** Anna Santarsiero, Ilaria Pappalardo, Gabriella Margherita Rosa, Isabella Pisano, Stefano Superchi, Paolo Convertini, Simona Todisco, Patrizia Scafato, Vittoria Infantino

**Affiliations:** 1 Department of Science, University of Basilicata, Viale dell’Ateneo Lucano 10, 85100 Potenza, Italy; 2 Department of Biosciences, Biotechnologies and Biopharmaceutics, University of Bari, Via Orabona 4, 70125 Bari, Italy

**Keywords:** chalcone, hepatocellular carcinoma (HCC), intrinsic pathway of apoptosis, mitochondria, BAX, BCL-2, caspase, Complex I

## Abstract

Hepatocellular carcinoma (HCC) is the most common type of liver cancer and the fourth cause of cancer-related deaths worldwide. Presently, a few drugs are available for HCC treatment and prevention, including both natural and synthetic compounds. In this study, a new chalcone, (*E*)-1-(2,4,6-triethoxyphenyl)-3-(3,4,5-trimethoxyphenyl)prop-2-en-1-one (ETTC), was synthesized and its effects and mechanisms of action over human hepatoma cells were investigated. Cytotoxic activity was revealed in HCC cells, while no effects were observed in normal hepatocytes. In HCC cells, ETTC caused subG1 cell cycle arrest and apoptosis, characterized by nuclear fragmentation. The activation of caspases 3/7 and 9, the increase in pro-apoptotic BAX, and the decrease in anti-apoptotic BCL-2 suggest the activation of the intrinsic pathway of apoptosis. ETTC mitochondrial targeting is confirmed by the reduction in mitochondrial membrane potential and Complex I activity together with levels of superoxide anion increasing. Our outcomes prove the potential mitochondria-mediated antitumor effect of newly synthesized chalcone ETTC in HCC.

## 1. Introduction

Hepatocellular carcinoma (HCC) is a primary liver cancer more common in males than in females and represents the fourth leading cause of cancer-related deaths worldwide (https://gco.iarc.fr/, assessed on 20 August 2022). HCC occurs most often in people suffering from chronic liver diseases, particularly cirrhosis, hepatitis B (HBV) or C (HCV) infections, and nonalcoholic fatty liver disease (NAFLD) [1]. The incidence is significantly relevant in the endemic areas of HBV infection, such as East Asia and sub-Saharan Africa, but is also rising in Western countries due to the greater increments in HCV infection, chronic alcoholic intake, and NAFLD [2]. Chronic liver exposure to viral hepatitis injury, NAFLD, or alcohol abuse produces numerous damages to the hepatocytes and creates a vicious cycle of cell death and regeneration. The consequent genomic instability is responsible for HCC initiation. The progressive accumulation of genetic alterations, such as somatic mutations, gene rearrangements, and alterations in copy number, and epigenetic changes lead to tumor progression and metastasis [3]. Therefore, the pathogenesis of HCC is a multistep process in which genetic, epigenetic, oxidative stress, inflammation, and immunity have key roles. Even if the main risk factors have been identified and some steps involved in hepatocarcinogenesis have been elucidated, the detailed molecular mechanisms related to the progression of HCC remain unclear; therefore, a better understanding of these mechanisms is urgently needed to develop new and effective therapeutic strategies, including new medications, and reliable prognostic biomarkers [4].

Although there have been advances in therapy and screening of high-risk patients, the improvement in prognosis has been minimal [5]. HCC can be treated with surgery (hepatectomy or liver transplantation) or ablation therapy, especially if it is diagnosed early. HCC is often diagnosed in advanced stages, so many patients require systemic therapy, such as chemotherapy or immune therapy [6]. Only a few drugs are available for HCC. Sorafenib was the first systemic drug approved by the US Food and Drug Administration for the treatment of advanced-stage HCC [7] and it was used as standard therapy between 2007 and 2016. Over the last few years, the progress in understanding the molecular mechanisms of tumorigenesis has allowed the development of new molecular targeted drugs, especially tyrosine kinase inhibitors (e.g., Cabozantinib) and monoclonal antibodies (e.g., Tremelimumab and Durvalumab) [5]. Recent research has shown that a single drug for HCC treatment may not be successful, so a major focus is on the use of combination therapy. At present, the regimens Atezolizumab + Bevacizumab and Durvalumab/Tremelimumab represent the first immune combination therapies more effective than Sorafenib in the treatment of advanced HCC [8,9].

The challenge for the management of advanced-stage HCC still remains and many efforts are directed towards the identification of new potential biomarkers. In this context, natural and synthetic biomolecules have demonstrated a potential anti-HCC effect taking into account their various biological and chemical properties [6,7].

Chalcones ((*E*)-1.3-diaryl-2-propen-1-ones) are naturally occurring compounds constituting the open-chain class of the flavonoid family. They are ubiquitous in edible and medicinal plants and have been isolated from different vegetables, fruits, and spices, especially from species belonging to the *Fabaceae*, *Asteraceae*, and *Moraceae* families. For example, licorice (*Glycyrrhiza glabra*) is an excellent source of isoliquiritigenin, isoliquiritin, neoisoliquiritin, licochalcone A, licochalcone B, echinatin, licuroside, and neolicuroside; apple fruit (*Malus domestica*) contains phloridzin, seboldin, and trilobatin; and tomatoes (*Solanum lycopersicum*) contain eriodictyol chalcone [10]. Natural chalcones have been found in petals, leaves, fruits, bark, and roots. However, being also intermediates in the biosynthesis of flavonoids, chalcones do not accumulate appreciably in most plants [11].

Natural and synthetic chalcones have aroused much interest for their broad spectrum of biological activities, including antioxidant, anti-inflammatory, immunomodulatory, antimicrobial, and anticancer properties [12,13,14,15]. Several of them have shown cytotoxicity against different cancer cell lines in low concentrations [16,17] through various mechanisms—depending on their specific structure—such as induction of apoptosis, cell cycle disruption, regulation of autophagy, and modulation of inflammatory mediators [18]. 

**Figure 1:**
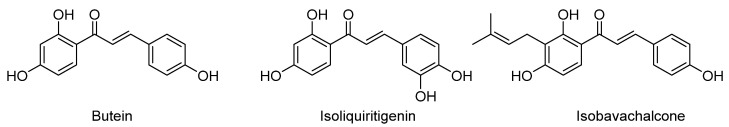
Structure of naturally occurring anticancer chalcones butein, isoliquiritigenin, and isobavachalcone.

Butein (3,4,2′,4′-tetrahydroxychalcone) is a naturally occurring chalcone, isolated from *Rhus verniciflua* stokes [19], with well-known anti-inflammatory, pain-relieving, anti-emetic, antioxidant, and anti-bacterial properties [20] in addition to marked anticancer activity [21]. Indeed, butein induces reactive oxygen species (ROS) generation and apoptosis [22], attenuates angiogenesis [23], controls cell invasion/metastasis, and reduces inflammation by inhibiting the nuclear factor-kappa B (NF-κB) signal pathway [24,25]. Moreover, in HCC, butein causes G2/M phase cell cycle arrest [23]. In addition to butein, isoliquiritigenin (isolated from licorice roots) and isobavachalcone (predominantly found in species of the *Fabaceae* and *Moraceae* families) (Figure 1) are two other natural chalcones with noticeable antitumor activity against various cancers [18].

The success of naturally occurring chalcones with potential therapeutic application for many types of cancer has encouraged many efforts directed at developing novel synthetic and chemically diverse chalcones acting with the same identified mechanism of natural chalcones [26]. Analysis of the molecular modifications of the structure responsible for better physicochemical and biological properties of chalcones has revealed that the manipulation of both aryl rings or their replacement with heteroaryl moieties or transformation in chalcone hybrids are the most effective modifications [15,27]. Several studies highlight that methoxy substitutions on both aryl rings and, in general, an increase in compound lipophilicity have significant effects on anticancer and other activities, influencing cell permeation as well as pharmacodynamic interactions with biological targets [18,28,29].

Starting with these considerations, we synthesized the new chalcone ((*E*)-1-(2,4,6-triethoxyphenyl)-3-(3,4,5-trimethoxyphenyl)prop-2-en-1-one) (ETTC), and we evaluated its effect on modulating the function of HCC cells for a future improvement in the HCC treatment strategy.

## 2. Materials and Methods

### 2.1. Synthesis of (E)-1-(2,4,6-triethoxyphenyl)-3-(3,4,5-trimethoxyphenyl)prop-2-en-1-one (ETTC) 

Synthesis of ETTC is detailed in the Supplementary Material.

### 2.2. Cell Culture 

The human HCC cell line HepG2 (European Collection of Authenticated Cell Cultures (ECACC)) was cultured in Dulbecco’s modified Eagle’s medium (DMEM) supplemented with 10% (*v*/*v*) fetal bovine serum, 2 mM L-glutamine, and 1% penicillin/streptomycin (Sigma-Aldrich, St Louis, MO, USA). Human hepatocytes (HH, Lonza, Walkersville, MD, USA) were maintained in hepatocyte culture medium (Lonza) according to manufacturer’s instructions. Cells were grown at 37 °C in a humidified 5% CO_2_ atmosphere. 

### 2.3. Isolation of Mitochondria

About 2 × 10^7^ HepG2 cells were harvested using trypsin and washed with phosphate buffer (PBS). All procedures were carried out at 0–4 °C. The HepG2 cells were suspended in 900 μL of 0.1× buffer A (50 mM MgCl_2_, 250 mM NaCl, 350 mM TRIS, pH 7.8), put on ice for 2 min, and homogenized by 20 passes in a tight-fitting glass/Teflon power-driven Potter-Elvehjem homogenizer. To the homogenate, 100 μL of buffer A was added and the mixture was centrifuged at 1600× *g* for 15 min. The pellet was resuspended in buffer A, rehomogenized, and centrifugated as before. The supernatant was transferred into a new tube and mitochondria were pelleted by centrifugation (12,000× *g*/10 min). The resulting mitochondrial pellet was resuspended in 100 μL of 320 mM sucrose, 1 mM EDTA, and 10 mM TRIS pH 7.4. The protein concentrations of the mitochondrial preparations were determined by Bradford protein assays (Bio-Rad Laboratories Inc., Hercules, CA, USA). The mitochondrial fraction can be stored at −80 °C. 

### 2.4. Cell Viability Assay

HepG2 (4 × 10^3^) and HH (2 × 10^3^) cells were seeded in a 96-well plate and allowed to attach overnight. The day after, cells were treated with DMSO (negative control), ETTC, or butein at 0.1, 1, 2, and 5 μM. The effect on cell proliferation was determined after 72 h by a CellTiter-Glo^®^ 2.0 Cell Viability Assay (Promega, Madison, WI, USA) as previously reported [30]. The viability of HepG2 cells was also evaluated 24 and 48 h post-ETTC treatment. CellTiter-Glo^®^ 2.0 Reagent (100 μL) was added to cells and mixed for 2 min on an orbital shaker to ensure cell lysis. Luminescence was recorded by a plate reader (GloMax, Promega) at the end of a 10 min incubation at room temperature. 

### 2.5. Cell Cycle Analysis

For cell cycle analysis, we adapted the procedure described in [31]. Briefly, HepG2 cells were seeded in a 6-well culture plate (2 × 10^6^ cells/well) and treated with 2 µM ETTC or an equal volume of DMSO (used as solvent). After 48 h, cells were collected, washed twice with cold PBS, fixed in EtOH 70% at −20 °C, and treated with 100 µg/mL RNase A. Finally, 50 µg/mL propidium iodide was added and the mixture was incubated for 30 min at room temperature. DNA content was detected using an Attune Nxt Acoustic Focusing Cytometer (Thermo Fisher Scientific, Waltham, MA, USA) equipped with a blue laser (488 nm). The percentage of cells in the subG1 phase, G1 phase, the S phase, and the G2 phase was analyzed using FlowJo™ 10.4 Software.

### 2.6. DAPI Staining

HepG2 cells (2 × 10^5^) were treated with 2 µM ETTC or DMSO for 24 h. Nuclear morphological changes were investigated by using 4′,6-diamidino-2-phenylin-dole (DAPI) staining [32]. Cells were washed with PBS and incubated with 300 nM DAPI for 10 min. Images were acquired with the Evos Floid Cell Imaging Station (Thermo Fisher Scientific). 

### 2.7. Caspase Assays

HepG2 cells (2 × 10^4^) were seeded in a 96-well plate and treated with 2 µM ETTC or DMSO. At different times after ETTC addition, the medium was removed and the activity of caspase 3/7 or 9 was determined by using a Caspase-Glo^®^ 3/7 or 9 assay kit (Promega), respectively, as previously described [33]. Luminescence was determined by a GloMax plate reader.

### 2.8. Real-Time PCR

HepG2 cells (1 × 10^6^) were seeded in a 6-well plate and treated with 2 µM ETTC or DMSO (control). Total RNA was extracted with an RNeasy Mini Kit (Qiagen, Venlo, The Netherlands). An iScript™ cDNA Synthesis Kit (Bio-Rad Laboratories) was used to obtain cDNA. Real-time PCR experiments were performed on a 7500 Fast Real-Time PCR System (Thermo Fisher Scientific) by using specific TaqMan Gene Expression Assays (Thermo Fisher Scientific) for human BCL-2 (Hs00608023_m1), BAX (Hs00180269_m1), and β-actin (4326315E–ID Hs01060665 ACTB—VIC—assay spans exons 2 and 3—amplicon length 63 bp). BCL-2 and BAX transcript levels were normalized against β-actin expression levels. Data were analyzed as previously described [34] according to the ΔΔct method. 

### 2.9. Mitochondrial Mass Analysis

HepG2 cells were seeded in a 24-well plate at a density of 2 × 10^5^ cells/well and treated with 2 µM ETTC or DMSO for 24 h. Mitochondrial mass was monitored using the MitoTracker^®^ Green FM (MTG, Thermo Fisher Scientific) fluorescence probe [35] following the manufacturer’s instructions. In brief, the medium was removed and a prewarmed (37 °C) staining solution containing 25 nM MTG probe was added. After 30 min of incubation at 37 °C in the dark, cells were washed with PBS and analyzed by the Evos Floid Cell Imaging Station. For quantitative analysis, the fluorescence was measured by using a GloMax plate reader (Promega). Images were also analyzed by the Mitochondria Analyzer tool (https://github.com/AhsenChaudhry/Mitochondria-Analyzer, accessed on 6 November 2022).

### 2.10. Mitochondrial Membrane Potential Analysis

HepG2 cells (2 × 10^5^) were seeded in a 24-well plate and treated with 2 µM ETTC, 2 μM FCCP, or DMSO for 24 h. Changes in mitochondrial membrane potential were monitored using MitoTracker™ Red CMXRos (MTR, Thermo Fisher Scientific) fluorescent dye that labels mitochondria in a manner dependent on the membrane potential [36], thus giving an indication of mitochondrial stress [32]. At the end of the treatments, the medium was removed and a prewarmed (37 °C) solution of 25 nM MTR was added. Following a 30 min incubation at 37 °C in the dark, cells were washed with PBS and visualized by the Evos Floid Cell Imaging Station (magnification 40×). Image analysis was performed with ImageJ software (https://imagej.nih.gov/ij/index.html, NIH, accessed on 30 November 2022) to quantify the intensity of mitochondrial red within the treated and control cells. 

### 2.11. Mitochondrial Complex I Activity Assay

Mitochondrial Complex I (NADH: CoQ, oxidoreductase) activity was determined on isolated mitochondria by measuring the oxidation of NADH. Briefly, 100 μg of mitochondria were added to a phosphate buffer (KH_2_PO_4_, 20 mM pH 7.4) containing 1 mM sodium azide, 10 mg/mL bovine albumine in 10 mM EDTA, pH 7.4, and 0.2 mM NADH. The reaction was started by adding 50 μM coenzyme Q1 and the assay was carried out using a spectrophotometer (Multiskan Sky, Thermo Fisher Scientific) by reading at 25 °C the decrease in NADH absorbance at 340 nm for at least 2 min. 

As control, the rotenone-sensitive Complex I activity was measured by adding 5 μM of rotenone and by following the slope change for 2 min. 

Complex I activity was calculated as nanomoles per minute per milligram of protein and normalized by the citrate synthase activity to minimize the effects of variability of mitochondrial numbers of cell cultures.

### 2.12. Mitochondrial Citrate Synthase Activity Assay

Citrate synthase (CS) activity was measured by the colorimetric method based on the reaction between CoA-SH, produced by the reaction catalyzed by CS, and 5, 5′-dithiobis-2-nitrobenzoic acid (DTNB) to form 5-thio-2-nitrobenzoic acid (TNB), which exhibits an intense absorbance at 412 nm. In brief, the assay mixture was formed of a medium containing TRIS-HCl buffer (mM pH 7.4) EDTA 0.5 mM, TRITON X100 0.1%, acetyl CoA 0.1 mM, oxalacetate 0.5 mM, and DTNB 0.1 mM. The reaction was started by adding 50 μg of mitochondria to the medium and the absorbance was measured at 412 nm at 25 °C with a spectrophotometer (Multiskan Sky, Thermo Fisher Scientific).

### 2.13. Mitochondrial Superoxide Analysis

HepG2 cells were seeded in a 24-well plate at a density of 2 × 10^5^ cells/well and treated with 2 µM ETTC or DMSO for 24 h. The production of the mitochondrial superoxide anion was assessed by the MitoSOX™ Red mitochondrial superoxide indicator (MS, Thermo Fisher Scientific), as previously described [33]. Cells were stained with 5 μM MS for 45 min at 37 °C in the dark, washed with PBS, and analyzed by the Evos Floid Cell Imaging Station (magnification 40×). Image analysis was performed with ImageJ.

### 2.14. Statistical Analysis 

Results are presented as mean values ± standard deviation (SD) of at least three independent experiments run in triplicate. Statistical analysis was performed by Student’s *t*-test or one-way ANOVA and, where indicated, a Tukey test was used as post-hoc test to compare differences between the means of samples and relative controls. According to the *p*-value, differences were considered as significant (*p* < 0.05), very significant (*p* < 0.01), or highly significant (*p* < 0.001).

## 3. Results

### 3.1. Design and Synthesis of Chalcone ETTC

**Scheme 1:**
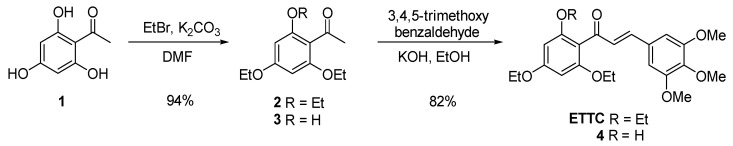
Synthesis of chalcones ETTC and 4.

As reported above, lipophilicity enhancement, in particular by adding methoxy groups on both aryl rings, has remarkable effects on chalcones’ anticancer activities. Therefore, we believed it interesting to prepare the new polyalkoxy-substituted chalcone ETTC (Scheme 1) in order to evaluate its activity on HepG2 cells. In this chalcone, etherification of all the phenolic functions imparts a higher lipophilic character to the molecule. Moreover, taking into account that ethoxy groups increase the molecular lipophilicity [28] and increase steric hindrance with respect to the methoxy one, three ethoxy groups were inserted on the 2′,4′,6′positions of the benzoyl ring, in which the substituent effect is more relevant for bioactivity. Accordingly, chalcone ETTC was prepared in high overall yield by alkylation of commercially available 2,4,6-trihydroxyacetophenone **1** with an excess of bromoethane, followed by classical Claisen–Schmidt condensation with 3,4,5-trimethoxybenzaldehyde (Scheme 1). The similar chalcone **4** was also prepared by the same procedure, starting from the 2-hydroxy acetophenone **3**, which itself was obtained by partial alkylation of acetophenone **1** with 2.0 equiv. of bromoethane. For both steps, experimental procedures based on principles of economics and green chemistry were used [37], avoiding the use of a large quantity of solvents, and avoiding high-solvent and time-demanding chromatographic purifications (see Supporting Information for details). This makes the synthetic procedure easily reproducible even on a large scale.

**Figure 2:**
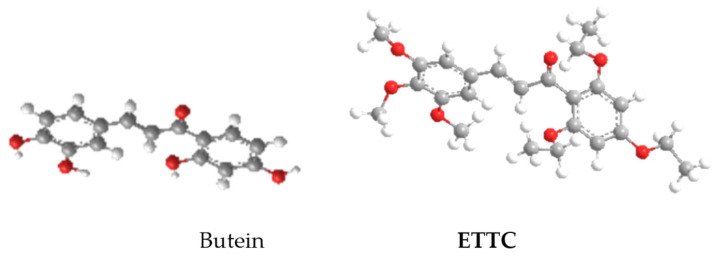
3D structure of butein and ETTC.

Furthermore, conformational analysis of ETTC revealed that the insertion of ethoxy substituents in both ortho-positions gave rise to significant effects on its three-dimensional structure. In fact, in constrast to butein and most chalcone derivatives, which are fully planar, in chalcone ETTC, the benzoyl ring is almost orthogonal with respect to the plane of the enone moiety, due to steric repulsions between the two ortho-ethoxy substituents and the carbonyl. In Figure 2, the 3D structure of totally planar butein is compared with that of ETTC.

### 3.2. ETTC Reduced HepG2 Cell Viability

**Figure 3:**
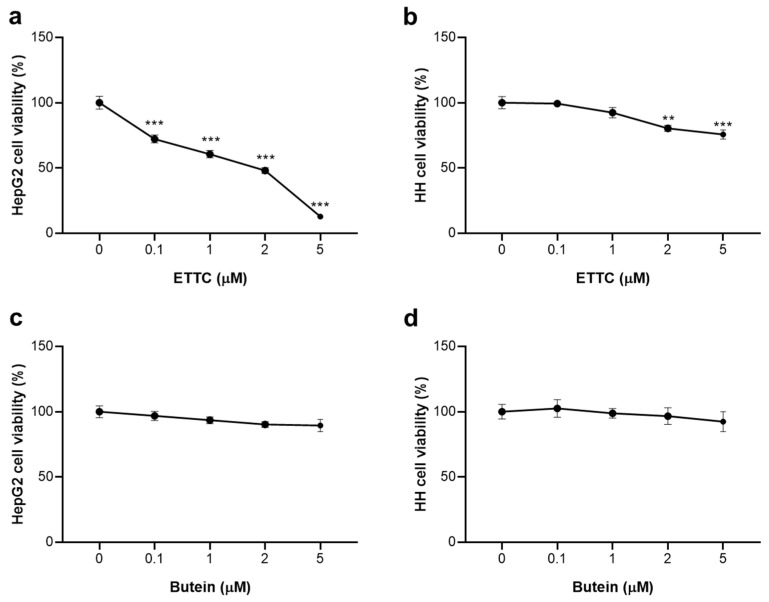
Effects of ETTC and butein on viability of HepG2 and HH cells. HepG2 and HH cells were treated with vehicle DMSO (0) or ETTC (**a**,**b**) or butein (**c**,**d**) at the indicated concentrations (0.1, 1, 2, and 5 µM). Cell viability was assessed after 72 h exposure. Data are expressed as mean values ± SD of three separate experiments and were analyzed by one-way ANOVA followed by Tukey’s post-hoc test. Where indicated, differences were significant (** *p* < 0.01, *** *p* < 0.001 vs. control (0)).

HepG2 and HH cells were treated with the new synthesized chalcone ETTC at different concentrations (0.1, 1, 2, and 5 μM) and the effects on cell viability were determined after 24, 48, and 72 h. After 24 h, 1 and 2 µM ETTC caused a slight impairment in HepG2 cell viability, whereas a marked decrease of about 30% was observed at 5 µM (Figure S1a). At 48 h, the same reduction of 30% was recorded at 1 μM; ETTC affected cell proliferation in a dose-dependent manner which was severely impaired at 5 μM with only 40% viable cells (Figure S1b). Instead, at 72 h, ETTC caused a significant reduction in HepG2 cell viability already at the lowest 0.1 µM concentration. A decrease of about 50% at 2 μM (IC_50_ = 2.65 μM), up to a full citotoxicity at the highest 5 μM concentration (Figure 3a), was observed. The viability of HH was slightly impaired at 72 h only at the higher concentrations (2 and 5 µM) following the treatment with chalcone ETTC (Figure 3b). 

The reference chalcone butein, on the other hand, showed little or no toxic effect in both cell lines (Figure 3c,d). Similar results were obtained in U937 cancer cells and the HEK293 non-tumor cell line (Figure S2). ETTC reduced U937 cell viability in a dose-dependent manner up to 5 μM (Figure S2a), while in HEK293 cells, a minimal cytotoxic effect was observed only at 2 and 5 μM (Figure S2b). Moreover, the presence of the three ethoxy groups on the benzoyl ring increased the selectivity of ETTC for cancer cells. In fact, we observed that the analogous chalcone 4, having a hydroxyl group in position 2 of the benzoyl ring in place of the ethoxy one and prepared for comparative purposes, exhibited almost the same cytotoxic effect to ETTC on HepG2 cells, but was significantly toxic in HH (Figure S3). 

Our results clearly evidenced the selectivity of ETTC for cancer cells, namely HepG2 and U937. The IC_50_ value for ETTC in HepG2 cells was calculated as being equal to 2.65 μM and we have chosen the sub-lethal concentration of 2 μM for all subsequent experiments.

### 3.3. ETTC Caused subG1 Cell Cycle Arrest and Nuclear Fragmentation in HepG2 Cells

**Figure 4:**
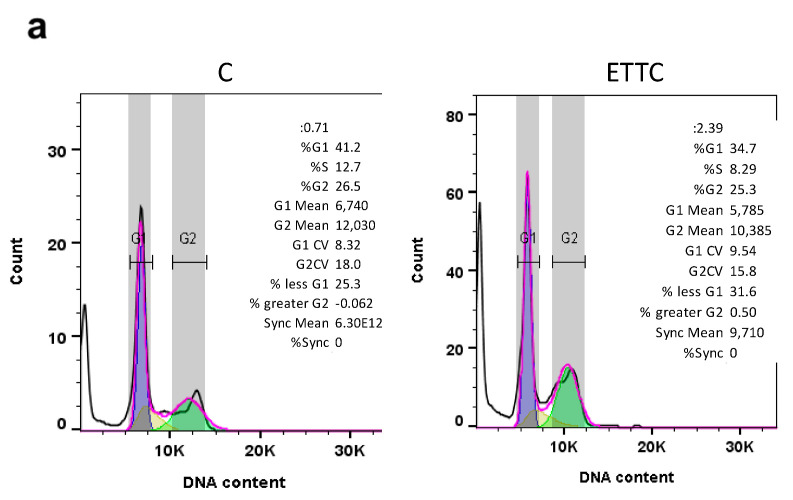

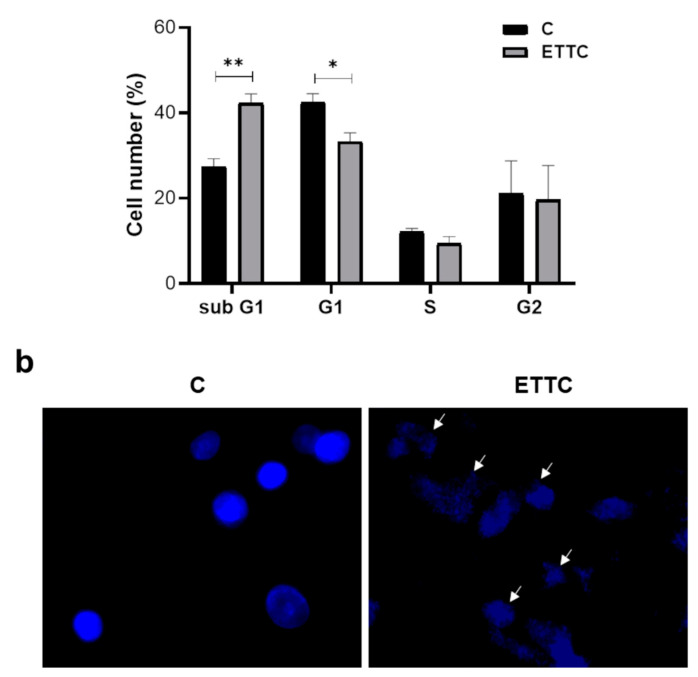
Effect of ETTC on cell cycle progression and nuclear morphology of HepG2 cells. (**a**) HepG2 were treated with ETTC and analyzed by flow cytometry after 48 h. The graph containing the percentage ± SD of cells in each phase of the cell cycle (G1:blue; S: yellow; G2: green; pink line represents the optimal histogram deduced by the FlowJo Cell Cycle Analysis algorithm) was obtained from three independent experiments. Statistical analysis was performed by Student’s *t*-test. Where indicated, differences were significant (* *p* < 0.05, ** *p* < 0.01). (**b**) Following 24 h treatment with DMSO (C) or ETTC, HepG2 cells were stained with DAPI and visualized under a fluorescence microscope (magnification 20×). Arrows indicate condensed nuclei of ETTC-treated HepG2 cells.

To clarify the molecular mechanism by which ETTC affects cell viability, we have studied the cell cycle. We then performed flow cytometry analysis after a 48 h treatment of HepG2 cells with ETTC at a concentration of 2 µM. Exposure of HepG2 cells to chalcone resulted in subG1 arrest compared to untreated controls. Forty-eight hours after ETTC addition, we observed a significant cell number increase in the subG1 phase by about 50% and a decrease in the G1 phase as well as in S phase when compared to untreated cells (Figure 4a). 

Staining with DAPI also revealed cellular apoptosis. In the presence of ETTC, HepG2 cells were undefined, less luminous, and characterized by blue, fluorescent dots representative of condensed and fragmented chromatin and membrane blebs. On the contrary, control cells had round nuclei and were stained with a uniform blue (Figure 4b). Together, the data point out that chalcone ETTC induces a subG1 cell cycle arrest in hepatocarcinoma cancer cells.

### 3.4. ETTC Induced Mitochondria-Mediated Apoptosis in HepG2 Cells

**Figure 5:**
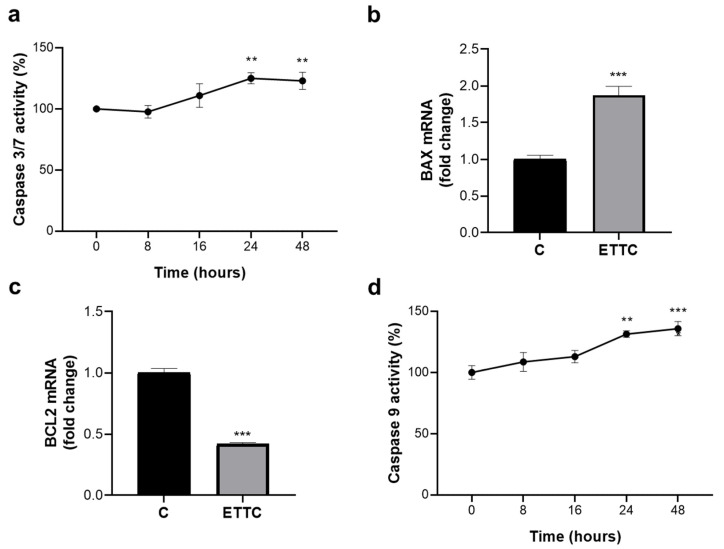
ETTC activates the intrinsic pathway of apoptosis. (**a**) HepG2 cells were treated with DMSO (0) or ETTC for 8–16–24–48 h and assayed for caspase 3/7 activity. mRNA levels of BAX (**b**) and BCL-2 (**c**) were evaluated after 24 h treatment with ETTC. (**d**) Cells were treated as in (**a**) and the activity of caspase 9 was measured. In (**a**–**d**), mean values ± SD of three independent experiments performed in triplicate are shown, where indicated differences between samples and relative controls are significant (** *p* < 0.01, *** *p* < 0.001, one-way ANOVA followed by Tukey test (**a**,**d**) and Student’s *t*-test (**b**,**c**)).

Since, following treatment with ETTC, we observed a reduction in HepG2 proliferation marked by a cell cycle arrest and chromatin fragmentation, we decided to evaluate the activity of caspases linked to cell death by apoptosis. We focused on apoptotic caspases, in particular caspases 3/7 and 9. Figure 5a shows that ETTC induced the activation of the caspases 3/7 by increasing the activity by almost 25% following 24 h of treatment to remain so at 48 h. Since these preliminary results suggest that the cytotoxic effect of ETTC was mediated by the induction of apoptosis, we wondered if the intrinsic pathway, the most frequently deregulated type of cell death in tumors, was involved in [38]. To this end, we evaluated the expression levels of anti-apoptotic BAX and pro-apoptotic BCL-2. The members of the BCL-2 protein family are considered “apoptotic switches” [39,40] in which some proteins, such as BCL-2, show an anti-apoptotic function, and others, such as BAX, show a pro-apoptotic function. The BAX/BCL-2 balance regulates both pro-apoptotic and anti-apoptotic intrinsic pathways by controlling the alteration of the permeability of the outer membrane of the mitochondria and thereby dictate the cellular fate [41]. ETTC induced a strong decrease in BCL-2 mRNA levels while BAX gene expression significantly increased (Figure 5b,c). As a consequence of the downregulation of BCL-2 and the overexpression of BAX, we observed the activation of caspase 9, the essential caspase required as an initiator for the mitochondrial pathway of apoptosis. Caspase 9 activity increased by 31.4 ± 2.6 % post-24 h treatment with ETTC (Figure 5d) and had similar rise following 48 h. Therefore, these findings indicate that chalcone ETTC’s antiproliferative effect in hepatocarcinoma cancer cells is mediated by the intrinsic apoptotic pathway. 

### 3.5. ETTC Caused Mitochondrial Dysfunctions in HepG2 Cells

**Figure 6:**
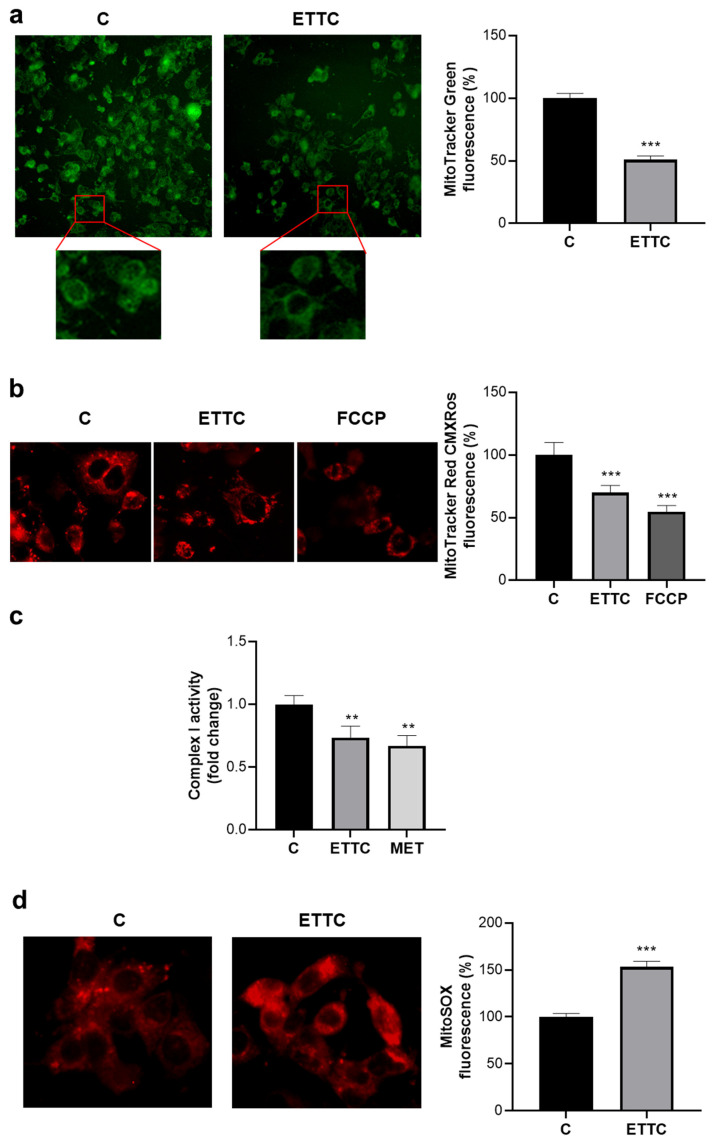
ETTC caused mitochondrial alterations. HepG2 cells, exposed to 2 μM ETTC or DMSO (**c**), were stained with MitoTracker Green FM (**a**), MitoTracker™ Red CMXRos (**b**), and MitoSOX Red Mitochondrial Superoxide Indicator (**d**). In (**b**), cells were also treated with 2 μM FCCP. Images were acquired under fluorescence microscope (magnification 20× in upper panel of (**a**), magnification 40× in lower panel of (**a**,**b**,**d**)). Images and relative bar graphs are representative of three independent experiments with similar results. (**c**) The activity of the Complex I was measured in isolated mitochondria from HepG2 cells treated as above (**a**,**b**,**d**) or with 2 μM Metformin (MET). The activity of Complex I was normalized against the activity of citrate synthase. The fold change was the ratio of enzyme activity from cells exposed to ETTC or Metformin with the activity relative to control. The data are representative of three independent experiments, and the bars are presented as means ± SD. (** *p* < 0.01, *** *p* < 0.001).

Taking into account the induction of the intrinsic apoptotic pathway following ETTC treatment, we focused on mitochondrial functionality by analyzing mitochondrial mass and mitochondrial membrane potential. Staining with MitoTracker Green showed a 50% ± 2.97% decrease in its intensity when HepG2 cells were treated with ETTC (Figure 6a), indicating a decrement in mitochondrial content, as the probe allows for mitochondrial mass quantification regardless of the mitochondrial membrane potential [42]. Furthermore, image analysis by the Mitochondria Analyzer tool revealed a significant lowering in total mitochondrial area following ETTC addition (Figure S4).

A reduction of about 30% was observed using MitoTracker Red CMXRos and this indicates a decrease in the membrane potential in the cells treated with ETTC (Figure 6b). As a positive control, we treated the HepG2 cells with FCCP, a potent uncoupler of mitochondrial oxidative phosphorylation [43].

Since it is known that redox balance and mitochondrial metabolism are both strongly dysregulated in liver diseases including HCC [44,45] we also evaluated mitochondrial ETC activity and superoxide levels as two important checkpoints for mitochondrial function.

The mitochondrial ETC is responsible for oxidation of reducing equivalents and recent evidence indicates that ETC supports cancer cell proliferation and resistance to cell death by maintaining NAD^+^ pool and redox balance, by promoting aspartate synthesis for purine and pyrimidine biosynthesis, and by inducing an adaptive mechanisms to hypoxia [46].

Isolated mitochondria from HepG2 treated with chalcone ETTC showed a decrease in the activity of Complex I, the first reducing equivalent entry point in ETC, compared to the activity of control cells, whereas the activity of other ETC complexes was not significantly affected (Figure S5). Notably, the effect of ETTC on Complex I activity was comparable to the decrease induced by metformin (Figure 6c), whose action mechanism implies the inhibition of Complex I. These results strengthen an altered mitochondrial functionality and an impairment to mitochondrial metabolism in HepG2 cells.

Evaluation of ETTC treatment on mitochondrial oxidative stress and superoxide radical production showed a significant increase in mitochondrial superoxide anion production, detected as an increase in MitoSOX fluorochrome targeting the mitochondria (Figure 6d). The overall results strongly suggest that chalcone ETTC acts through the intrinsic apoptotic pathway. Finally, to prove that the cytotoxic effects observed in HepG2 cells were strictly dependent on mitochondrial functionality impairment, we evaluated the effect of ETTC on normal hepatocytes. Figure S6 underlines that ETTC has no effect on mitochondrial mass (Figure S6a), mitochondrial membrane potential (Figure S6b), and superoxide anion production (Figure S6c) in HH cells. 

## 4. Discussion

In recent years, the main research focus for HCC treatment has been the development of a combination therapy, in which metabolic pathways are among the most important targets. The metabolic alterations are functional for cancer growth and metastasis and the understanding of metabolic reprogramming enables us to identify the key metabolism points on which to act to address metabolic cancer treatment. HCC is characterized by a global metabolic reprogramming in which the maintenance of redox balance and the rewiring of the tricarboxylic acid (TCA) cycle are crucial for cancer proliferation and survival [47]. New drugs that disrupt the metabolism of HCC cells and cause apoptosis by impairing mitochondrial function may then be an effective therapeutic strategy for this.

Natural and synthetic chalcones are a family of compounds which show many biological activities including antioxidant, anti-inflammatory, and anticancer activities and more chalcones have been approved for clinical treatment of different diseases [18]. Among natural chalcones, butein has aroused great interest based on its anticancer effect against a variety of tumors, such as colon cancer, liver cancer, and breast cancer [48], as a consequence of inhibition of proliferation, induction of apoptosis, and interaction with different molecular targets [49]. Butein is absorbed in the small intestine, due to its high solubility in basic pH, and undergoes methylation, glucuronidation, and sulfation by the liver [49]. Its high lipophilicity allows it to be absorbed in all tissues, but, at the same time, butein shows a low oral bioavailability caused by the reaction of methylation or glucuronidation in the small intestine. Furthermore, enterohepatic circulation is involved in the metabolism and excretion of butein and its derivatives [50]. Pharmacokinetic profiles demonstrate that, in plasma, butein binds to human serum albumin with hydrophobic interactions. The partition coefficient of butein in liposomes shows that it is efficiently distributed in liposomes, indicating that butein is able to permeate the cell membrane [51]. This prompted us to undergo this study aimed at finding a novel chalcone derivative with improved efficacy with respect to butein.

Recent research has shown that the specific positions of the hydroxyl and methoxy groups on the phenyl ring determine the anticancer properties of synthetic chalcones by increasing their cytotoxicity [18,52,53]. As a matter of fact, the newly synthesized polyalkoxylated chalcone ETTC exhibits a strong cytotoxicity for HepG2 cells and only a slight impairment to normal HH cell viability while, on the other hand, butein shows poor cytotoxicity for both cell lines. Notably, the IC_50_ of ETTC for HepG2 cells was 2.65 μM, much lower than that of other groups of synthetic chalcones [54,55,56]. In addition, 2-hydroxy-4,6-diethoxy chalcone 4 shows similar activity to ETTC with regard to HepG2 cells, confirming that a greater lipophilic character of the molecules increases their toxicity (ETTC and chalcone 4 vs. butein). However, unlike ETTC, chalcone 4 was found to be non-selective and very cytotoxic, even for normal HH cells. The only structural difference between the two molecules is the presence, in chalcone 4, of the hydroxyl group on one of the two ortho-positions of the benzoyl ring, in place of the ethoxy group present in ETTC. It is probable that this hydroxy group gives rise to hydrogen bonding with the carbonyl nearby, stabilizing a planar molecular structure, as in butein. On the contrary, the two ortho-ethoxy substituents in ETTC prevent the planar arrangement, giving rise to a repulsive steric effect with the carbonyl. Based on these considerations, it is possible to speculate that the selectivity of ETTC for HepG2 cancer cells could be related to the presence of the three ethoxy groups on the benzoyl ring and to the unusual spatial rearrangement of this chalcone that derives from it. The treatment of HepG2 with chalcone ETTC shows a subG1 cell cycle arrest with induction of apoptosis. Apoptosis is a type of physiological programmed cell death characterized by specific changes in cell morphology (i.e., nuclear fragmentation, chromatin condensation, blebbing, apoptotic bodies) and activation of caspases [57]. Caspases belongs to a family of endoproteases at the center of critical regulatory networks that control cell death [58]. ETTC induced nuclear fragmentation and the activation of caspases 3/7. The decrease in the BCL-2/BAX ratio, the increase in caspase 9 activity, and the reduction in mitochondrial membrane potential indicate an apoptosis induction via the intrinsic pathway. The mitochondrial alteration was confirmed by observing a decrease in mitochondrial mass and in ETC Complex I activity linked with an increase in superoxide levels. Mitochondria represents the primary source of intracellular reactive oxygen species which contribute to the apoptotic process by inducing lipid and protein oxidation and DNA damage [59]. 

In light of our results, the identification of the ETTC molecular target may aid in the development of other chalcones to trigger mitochondrial apoptosis more successfully. Our preliminary analyses suggest that the mechanism of action of ETTC involves inhibition of ETC Complex I as well for metformin, as proposed in [60]. The inhibition of Complex I leads to a decrease in O_2_ consumption and accumulation of NADH which, in turn, is an allosteric inhibitor of the TCA cycle. Dysregulations of the TCA cycle impair prolyl hydroxylases’ (PHD) activity, the enzymes responsible of destabilization of HIF-1α, a master regulator of functional adaption to hypoxia in cancer cells [61]. Indeed, a change in the NAD^+^/NADH ratio leads to inhibition of the α-ketoglutarate dehydrogenase complex (KCGDHC) and an increase in the levels of α-ketoglutarate (αKG). αKG is a co-substrate of PHD, the activity of which induces HIF-1α degradation and as a result, changes in the proliferation of cancer cells until cancer growth arrest occurs.

In conclusion, this study suggests that a sub-lethal concentration (2 μM) of ETTC induces apoptosis via the intrinsic pathway in HCC cells together with mitochondrial alterations involving Complex I function, the activity of which is decreased in the presence of chalcone ETTC. However, further studies are needed to elucidate the molecular mechanism of ETTC in major detail. Future investigations on the metabolism and bioavailability of ETTC in 3D cell models as well as in vivo investigations will be important in predicting ETTC use in clinical trials. Additionally, in vivo tests regarding ETCC toxicity in animal models might unveil the effect of this compound on tumor growth and explore its potential as a synergistic approach to the treatment of HCC.

## Data Availability

The data used to support the findings of this study are available from the corresponding authors upon request.

## Supplementary Materials

The following supporting information can be downloaded at: https://www.mdpi.com/article/10.3390/biomedicines10123120/s1, 1. Synthesis of ETTC and chalcone 4; 2. Cell culture; 3. Mitochondrial Electron Transport Chain Activity Assay; Figure S1: HepG2 cell viability 24 h and 48 h after ETTC treatment; Figure S2: Effect of ETTC on U937 and HEK293 cell viability; Figure S3: Effect of chalcone 4 on HepG2 and HH cell viability; Figure S4: Total mitochondrial area; Figure S5: Effect of ETTC on ETC complexes; Figure S6: Effect of ETTC on HH mitochondria. Reference [62] is cited in Supplementary Materials.
