# Supplementary material for: Mitochondrial Role in Intrinsic Apoptosis Induced by a New Synthesized Chalcone in Hepatocellular Carcinoma Cells
Source: Biomedicines. 2022 Dec 2;10(12):3120. doi: 10.3390/biomedicines10123120 (PMC9775964; doi:10.3390/biomedicines10123120)
Supplement: Supplementary file 1 [file biomedicines-10-03120-s001.zip › biomedicines-2058054-supplementary.pdf]

Supplementary Materials

S1. Synthesis of ETTC and chalcone 4

General Experimental Procedures: ^{1}H (400 MHz) and ^{13}C (100 MHz) NMR spectra were recorded in $CDCl_3$ on a Varian INOVA 400 spectrometer, using tetramethylsilane (TMS) as an internal standard. GC-MS analyses were performed on a HP-6890 gas-chromatograph equipped with a HP-5975 mass spectrometer detector and a HP-5MS capillary column. Analytical thin layer chromatography (TLC) was performed using silica gel 60 Macherey-Nagel sheets and visualized by ultraviolet radiation. Dimethylformamide (DMF) was dried by distillation over calcium hydride and stored under a nitrogen atmosphere. The other analytical grade solvents and commercially available reagents were used without further purification.

2,4,6-Triethoxyacetophenone **2**.
To a solution of 2,4,6-trihydroxyacetophenone **1** (168 mg, 1.0 mmol) in DMF (2.0 mL) potassium carbonate (660 mg, 4,78 mmol) and a solution of bromoethane (0.315 mL, 3.5 eq) in DMF (2.0 mL) were added in sequence. The mixture was stirred at room temperature, monitoring the progress of the reaction by TLC and GC analysis. When complete conversion was achieved (about 24h) the mixture was filtered under reduced pressure and the filtrate was poured in 10 mL of a water-ice mixture. The formed precipitate, corresponding to the desired product, was filtered under reduced pressure and washed with 2 mL of cold 5% NaOH and water, affording the pure triethoxyacetophenone **3** in 94% yield.
^{1}H-NMR (400 MHz, $CDCl_3$): ◎◎(ppm) 1.37 (t, J = 7.2 Hz, 6H), 1.41 (t, J = 6.8 Hz, 3H), 2.47 (s, 3H), 4.01 (m, 6H), 6.07 (s, 2H); ^{13}C-NMR (100 MHz, $CDCl_3$): ◎ (ppm) 14.6, 14.7, 32.5, 63.6, 64.2, 92.0, 114.3, 157.5, 161.4, 201.8; MS(EI): m/z 252 (M^+, 20), 237 (100), 209 (16), 181 (12), 153 (35), 69 (15), 43 (11).

*(E)-1-(2,4,6-triethoxyphenyl)-3-(3,4,5-trimethoxyphenyl)prop-2-en-1-one (**ETTC**).*
To a solution of 2,4,6-triethoxyacetophenone (**3**) (167 mg, 0.66 mmol) in 3 mL of ethanol a 60% aqueous KOH (2.4 mL) was added at room temperature. After 5 min, a solution of 3,4,5-trimethoxybenzaldehyde (233 mg, 1.8 eq.) in ethanol (3.5 mL) was added dropwise and the mixture was stirred, monitoring the conversion by TLC analysis. After 24h, ethanol was removed by distillation under reduced pression and the residue was quenched with water. A yellow precipitate was obtained, which was filtered under reduced pressure and washed with cold water. Recrystallization from 95% aqueous ethanol afforded the desired pure chalcone **1** with 82% yield.
^{1}H-NMR (400 MHz, $CDCl_3$): ◎ (ppm) 1.30 (t, J = 7.0 Hz, 6H), 1.44 (t, J = 7.0 Hz, 6H), 3.88 (s, 9H), 4.00 (q, J = 7.0 Hz, 4H), 4.06 (q, J = 7.0 Hz, 2H), 6.14 (s, 2H), 6.75 (s, 2H), 6.88 (d, J = 15.6 Hz, 1H), 7.25 (d, J = 15.6 Hz, 1H); ^{13}C-NMR (100 MHz, $CDCl_3$): ◎ (ppm) 14.6, 14.7, 56.1, 60.9, 63.6, 64.3, 92.2, 105.2, 128.7, 130.6, 143.8, 153.3, 158.1, 161.5, 194.3; MS(EI): m/z 430 (M^+, 75), 415 (17), 402 (100), 387 (53), 249 (29), 221 (29), 193 (36), 181 (48), 153 (41), 69 (17).

*2,4-diethoxy-6-hydroxyacetophenone (**3**).*

This compound was prepared in 76% yield, starting from **1** and 2.0 equiv. of bromoethane, following the same procedure used for acetophenone **2**.

^{1}H-NMR (400 MHz, $CDCl_3$) ◎ (ppm) : 1.40 (t ,J = 6.8 Hz, 3H), 1.47 (t, J = 7.2 Hz, 3H), 2.62 (s, 3H), 4.05 (m, 4H), 5.89 (s,1H), 6.02 (s, 1H), 14.04 (s, 1H); ^{13}C-NMR (100 MHz, $CDCl_3$)◎◎◎◎ppm) : 14.1, 14.5, 33.0, 63.8, 64.3, 91.5, 93.7, 105.9, 162.2, 165.4, 167.5, 203.1.

*(E)-1-(2,4-diethoxy-6-hydroxyphenyl)-3-(3,4,5-trimethoxyphenyl)prop-2-en-1-one (**4**).*

This chalcone was prepared in 93% yield, starting from acetophenone **3**, following the same procedure applied for **ETTC**.

^{1}H-NMR (400 MHz, CDCl3) ◎ (ppm) : 1.43 (t, J = 7.0 Hz, 3H), 1.59 (t, J = 6.8 Hz, 3H), 3.90 (s, 9H), 4.07 (q, J = 6.8 Hz, 2H), 4.11 (q, J = 7.0 Hz, 2H) , 5.94 (s, 1H), 6.09 (s, 1H), 6.84 (s, 2H), 7.72 (d, J = 15.4 Hz, 1H), 7.99 (d, J = 15.4, 1H), 14.5 (s, 1H); ^{13}C-NMR (100 MHz, CDCl3) ◎ (ppm) : 14.6, 15.01, 56.1, 61.0, 63.9, 64.4, 92.2, 94.2, 105.4, 106.1, 126.9, 131.2, 142.4, 153.4, 161.8, 165.6, 168.6, 192.3.

S2. Cell culture

Human monoblastic leukemia U937 cells (ICLC HTL94002-Interlab Cell Line Collection) were maintained in suspension in RPMI 1640 medium supplemented with 10% fetal bovine serum, 2 mM L-glutamine, 100 U/mL penicillin, and 100 μg/mL streptomycin. Human Embryonic Kidney 293 cells (HEK293, American Type Culture Collection (ATCC) (Manassas, VA, USA)) were grown in DMEM medium supplemented with 10% fetal bovine serum, 2 mM L-glutamine, 100 U/mL penicillin, and 100 μg/mL streptomycin. All cells were maintained at 37 °C in a humidified atmosphere of 95% air and 5% CO_2.

S3. Mitochondrial Electron Transport Chain Activity Assay

Mitochondrial Electron Transport Chain (ETC) complexes (Complex II, III and IV) activity was carried out on isolated mitochondria (100 μg) using a spectrophotometer (Multiskan Sky, Thermo Fisher Scientific).

Complex II (succinate: CoQ 1 oxidoreductase) was assayed by following the reduction of the ubiquinone coenzyme Q1 at 280 nm at 25°C. The assay buffer contained KH_2PO_4, 50 mM pH 7.4, 1 mM KCN, 10 mM sodium succinate, 10 μM antimycin A, and 5 μM rotenone. The reaction was started by adding 50 μM coenzyme Q1. Complex II activity was measured by following the slope change for at least 3 min.

The activity of Complex III (ubiquinonol: cytochrome c oxidoreductase) was measured at 550 nm following the reduction of cytochrome c by using, as electron donor, reduced decylbenzylquinone (DB·H2), an analogue of endogenous CoQ10. The assay reaction was composed by a phosphate buffer (KH_2PO_4 50 mM, pH 7.4), 1 mM n-dodecylmaltoside, 1 mM KCN, 5 μM rotenone, 0.1% (w/v) BSA and 0.1 mM DB·H2.

The reaction assay was initiated by adding cytochrome c 15 μM and the absorbance was read for 3-5 min. The fully reduction of cytochrome c was measured by adding L -ascorbic acid and a final absorbance reading was made.

The Complex IV (cytochrome c oxidase) assay determined the oxidation of reduced cytochrome c with H_2O formation by measuring the absorbance at 550 nm. Briefly, the reaction assay occurred in a buffer KH_2PO_4 50 mM, pH 7.4, by adding reduced cytochrome c 15 μM The absorbance was immediately read at 550 nm and the complete oxidation of cytochrome c was measured by adding 1 mM $K_3Fe(CN)_6$.

The activity of Complex II, III and IV was determined according literature data [1]. All electron transport chain complexes activities were normalized by the citrate synthase activity to minimize the effects of variability of mitochondrial numbers of cell culture.

Reference

1. Frazier, A. E. & Thorburn, D. R. Biochemical Analyses of the Electron Transport Chain Complexes by Spectrophotometry. in 49–62 (2012). doi:10.1007/978-1-61779-504-6_4.

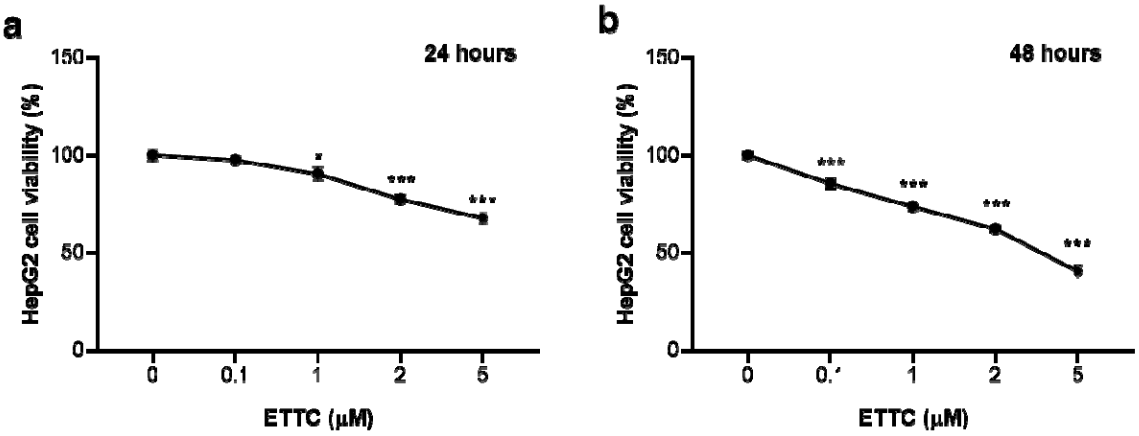

Figure S1. HepG2 cell viability 24 h and 48 h after **ETTC** treatment. HepG2 cells were treated with vehicle DMSO (0) or **ETTC** at the indicated concentrations (0.1, 1, 2 and 5 μM). Cell viability was assessed after 24 h (a) or 48 h (b) exposure. Data are expressed as mean values ± SD of three independent experiments and were analyzed by one-way ANOVA followed by Tukey post hoc test. Where indicated, differences were significant (* $p < 0.05$, *** $p < 0.001$ *vs*. control (0)).

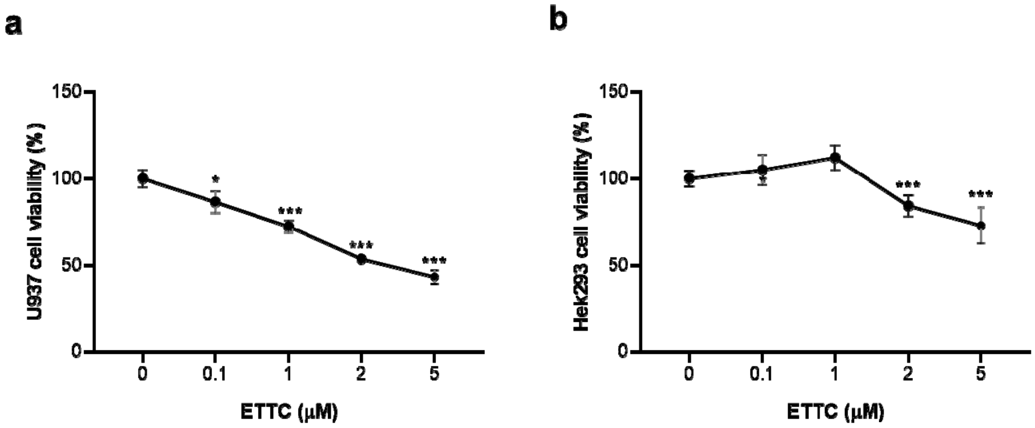

Figure S2. Effect of **ETTC** on U937 and HEK293 cell viability. U937 (**a**) and HEK293 (**b**) cells were treated with vehicle DMSO (0) or **ETTC** at the indicated concentrations (0.1, 1, 2 and 5 μM). Cell viability was assessed after 72 h exposure. Data are expressed as mean values ± SD of three separate experiments and were analyzed by one-way ANOVA followed by Tukey post hoc test. Where indicated, differences were significant (* $p < 0.05$, *** $p < 0.001$ *vs*. control (0)).

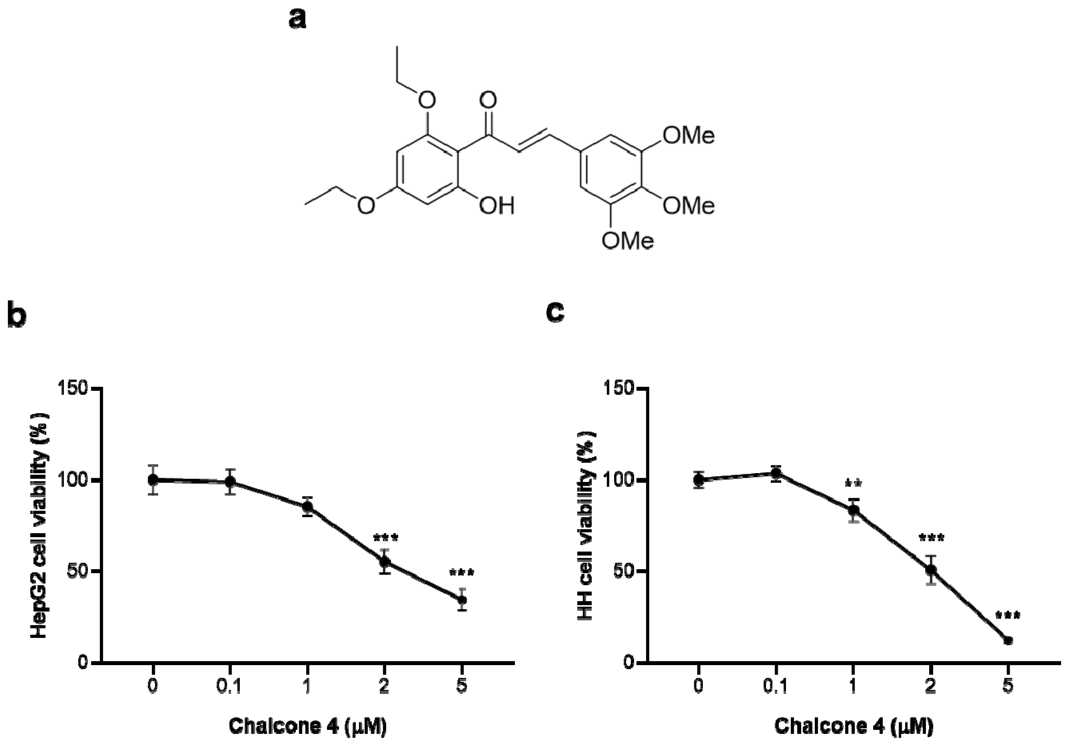

Figure S3. Effect of chalcone 4 on HepG2 and HH cell viability. (**a**) Molecular structure of chalcone **4**. (**b-c**) HepG2 (**b**) and HH (**c**) cells were treated with vehicle DMSO (0) or chalcone **4** at the indicated concentrations (0.1, 1, 2 and 5 μM). Cell viability was assessed after 72 h exposure. Data are expressed as mean values ± SD of three separate experiments and were analyzed by one-way ANOVA followed by Tukey post hoc test. Where indicated, differences were significant (** $p < 0.01$, *** $p < 0.001$ with respect to control (0)).

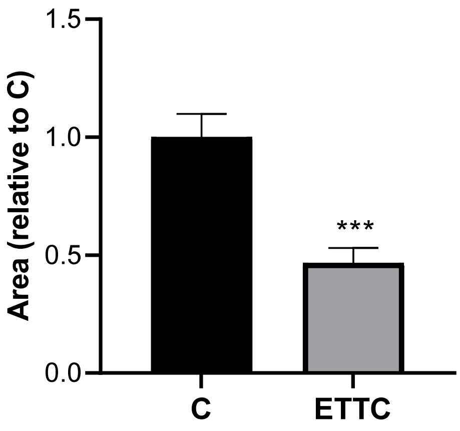

Figure S4. Total mitochondrial area. HepG2 cells were treated with vehicle control (DMSO, C) or 2 μM **ETTC** and total mitochondrial area was quantified. Bars represent mean values ± SD of three experiments. Data were analyzed by *Student's t-test* and differences were significant (*** $p < 0.001$).

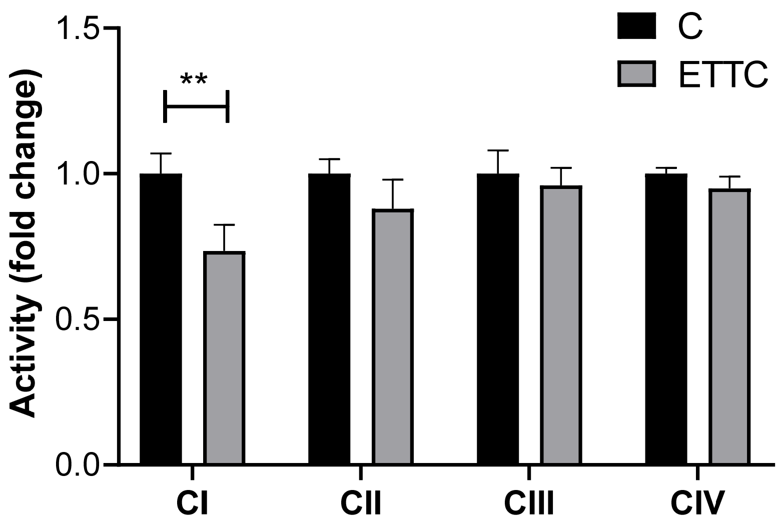

Figure S5. Effect of **ETTC** on ETC complexes. The activity of ETC complexes was measured on isolated mitochondria from HepG2 cells in the presence or absence of 2 μM **ETTC**. All activities were normalized against the activity of citrate synthase. The fold change was the ratio of enzyme activity from cells exposed to ETTC versus the activity relative to control. The data are representative of three independent experiments, and the bars are presented as means ± SD (** $p < 0.01$).

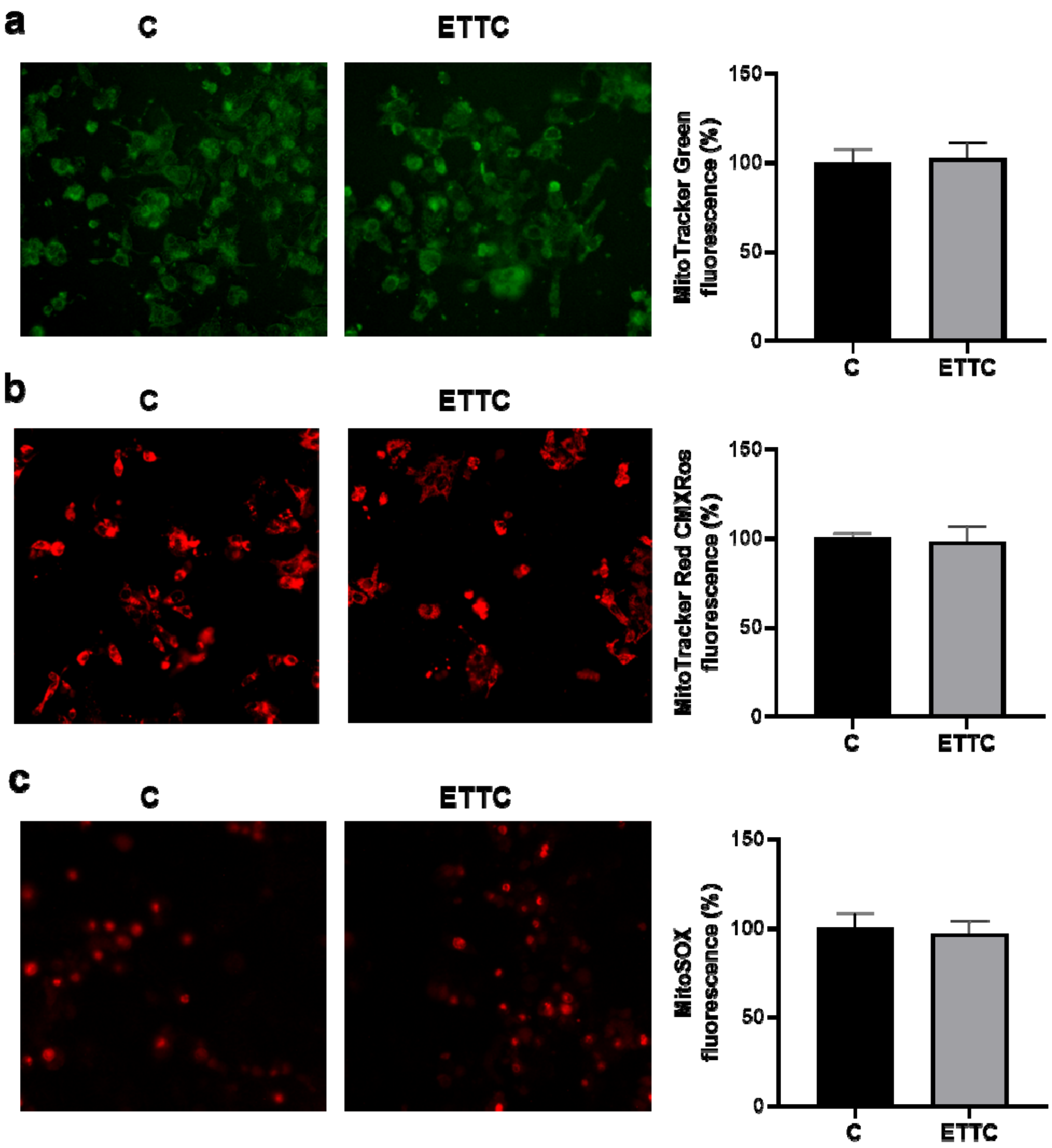

Figure S6. Effect of **ETTC** on HH mitochondria. HH cells, exposed for 24 h to DMSO (C) or **ETTC**, were stained with MitoTracker Green FM (a), MitoTracker™ Red CMXRos (b), and MitoSOX Red Mitochondrial Superoxide Indicator (c) and visualized under fluorescence microscope (magnification 20×). Images and relative bar graphs are representative of three independent experiments with similar results. Data were analyzed by *Student's t-test* and differences were not statistically significant.
